# Does financial transparency substitute corporate governance to improve stock liquidity? Evidence from emerging market of Pakistan

**DOI:** 10.3389/fpsyg.2022.1003081

**Published:** 2022-11-14

**Authors:** Shuaib Ali, Wu Zhongxin, Zahid Ali, Muhammad Usman, Yu Zhuoping

**Affiliations:** ^1^School of Management, Hainan University, Hainan, China; ^2^Department of Commerce and Management Sciences, University of Malakand Pakistan, Khyber Pakhtunkhwa, Pakistan; ^3^Division of Management and Administrative Sciences, UE, Business School, University of Education, Lahore, Pakistan

**Keywords:** corporate governance, stock liquidity, financial transparency, Pakistan, PCA

## Abstract

The aim of this study is to empirically analyze the impact of corporate governance on stock liquidity and the moderating role of financial transparency, through the lens of information asymmetry and agency theory. The sample consists of non-financial firms listed on the Pakistan stock exchange during the 2009–2019 period. The study used an instrumental variable approach and new corporate governance index, developed with principal component analysis, to demonstrate a relationship between corporate governance and stock liquidity. The results show a significant, positive relationship between the corporate governance index and stock liquidity, suggesting that well governed firms have high liquidity. To the best of our knowledge, this is the first finance study to investigate the moderating impact of financial transparency on the relation between corporate governance and stock liquidity. The results show that financial transparency, as measured by multiple proxies, has a negative moderating impact on the relationship between corporate governance and stock liquidity, suggesting that corporate governance in Pakistan is weak. Together, the results suggest that Pakistani firms use financial transparency as a substitute for corporate governance to improve stock liquidity. The results are robust to a series of endogeneity checks using alternative proxies of stock liquidity.

## Introduction

Stock liquidity is considered to be an important factor in the micro-structure of the economy and has been continuously addressed in the finance literature. Stock liquidity plays an important role in market development (Singh and Sharma, 2016), and high-premium markets are illiquid. Regulators or financial analysts improve liquidity by focusing on academic and professional concerns.

Corporate governance frameworks have been developed in response to deficiencies and scandals. The South Sea Bubble of 1700 was the first corporate governance collapse in England, while the stock market crash of 1929 was the first collapse in the United States. These crises exposed deficiencies and elicited regulations and procedures in both the United Kingdom and the United States. The well-known corporate scandals involving Enron, WorldCom, and Waste Management have similarly contributed to corporate governance reforms. The literature offers no agreed-upon definition of “corporate governance,” and this has been a subject of debate over the last several decades.

Effective corporate governance is necessary for active and professional stock markets. Strong corporate governance principles also lead to greater investor confidence in the market. Gilson (2000) argues that investment in stocks demands high corporate governance quality, and practical business information also requires effective corporate governance. Despite these claims, there is little empirical evidence for the positive association between high quality corporate self-governance and stock liquidity, although the quality of corporate governance is known to enhance stock liquidity in the U.S. (Chung et al., 2010).

The availability of accurate commercial information reduces information asymmetry and prevents misuse of accurate information. Financial transparency is essential to controllers, analysts and consumers of financial statements, as it encourages researchers to recognize the mechanisms and linked variables. Previous studies explore the mechanisms of financial transparency *via* agency and signaling theory of structural and principle perspectives (Leuz et al., 2003; Bassett et al., 2007).

This study uses a sample of non-financial firms listed on the Pakistan stock exchange during the 2009–2019 period. To the best of our knowledge, this is the first finance study to investigate the moderating effect of financial transparency on the relation between corporate governance and stock liquidity. It is also the first study to analyze the relationship between corporate governance and stock liquidity in Pakistan and the first study to establish new indexes for corporate governance using principal component analysis (PCA). Various number studies have been followed to develop the research question and describe the significance of the study, i.e., around 90 papers, including 60 from web of science and around 40 from Scopus.

Pakistan has highly concentrated firm ownership, with most firms being held by families. Most corporate boards are merely a “rubber stamp,” with the family holding the bulk of shares. Pakistani firms rely mostly on bank loans for financing. The public capital market has a more passive role in financing than in developed markets. As Pakistani firms rely much less on capital market financing than firms in developed countries, stock liquidity plays a different role in Pakistan. Furthermore, its capital market does not efficiently communicate information, but instead has weak financial transparency. This has resulted in information asymmetry and problems related to adverse selection. Therefore, the Pakistan market is significantly less liquid than the U.S. market.

The study has used an instrumental variable approach to develop a corporate governance index *via* PCA. The results show a significant, positive relationship between corporate governance and stock liquidity, suggesting that well governed firms have high liquidity. The evidence has found that under high financial transparency, the relationship between corporate governance and stock liquidity is weakened, suggesting that in Pakistan corporate governance is weak, causing firms to use financial transparency as a substitute for corporate governance to improve stock liquidity. The results are robust to a series of endogeneity checks using alternative proxies for stock liquidity.

**Figure 1:**
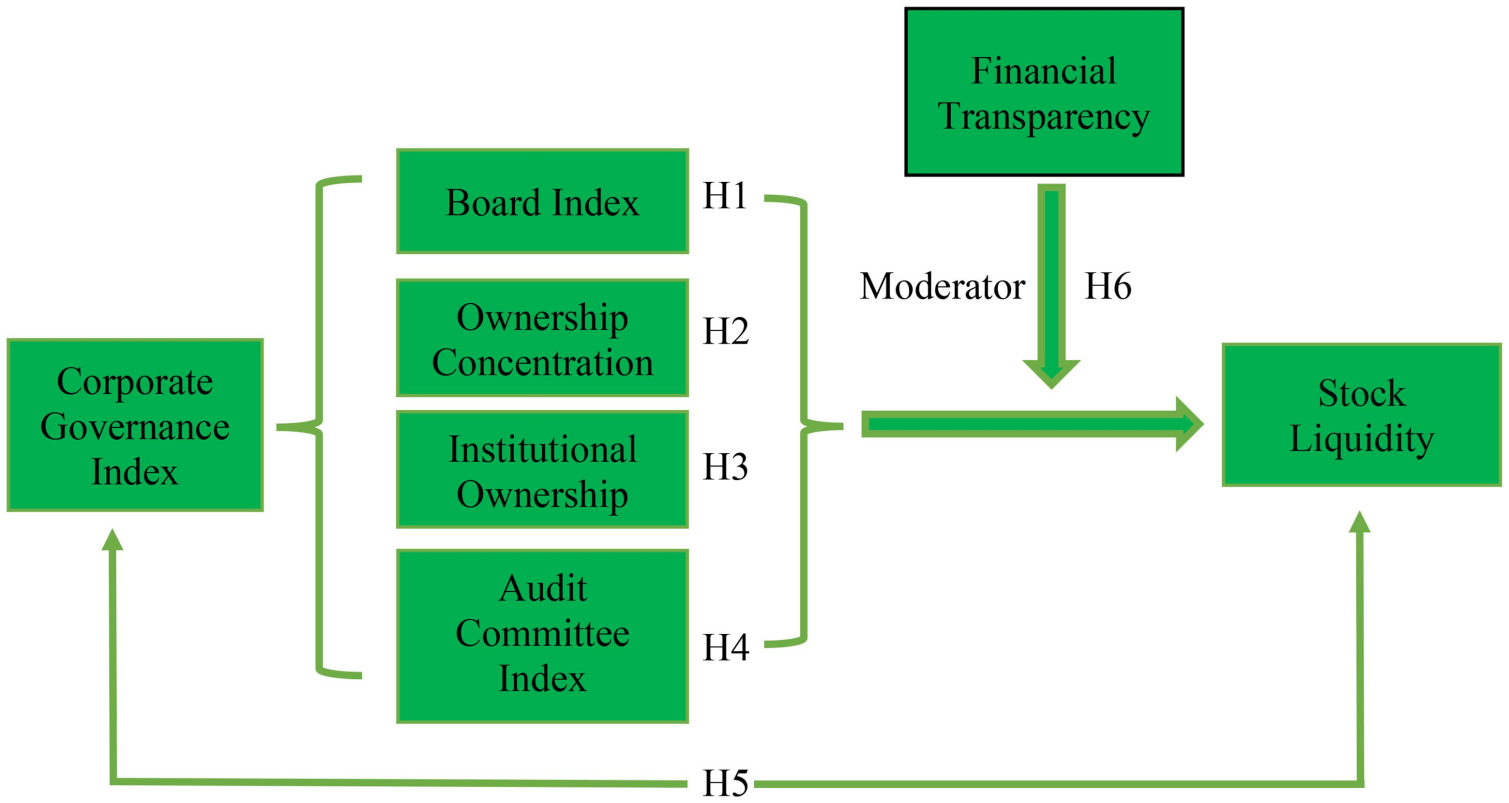
Conceptual framework.

The remainder of the paper is structured as follows. Section 2 provides a review of the relevant literature. Section 3 describes the hypothesis development. Section 4 explains the data and research design used to examine corporate governance and stock liquidity. Section 5 discusses the results of the study. Section 6 presents the conclusions, including limitations, future directions, and policy implications, and the conceptual framework is shown in Figure 1.

## Literature review and hypothesis development

### Board characteristics and stock liquidity

Corporate management is responsible for decision making and high-level regulation of a firm. Director independence is a much-discussed subject in corporate governance literature. Since Fama and Jensen (1983) established the value of a free and productive board of directors. The literature shows a relationship between female board members and both strong monitoring and the effective communication of information (Adams and Ferreira, 2009; Gul et al., 2011). Gul et al. (2011) also state that female directors expect higher monitoring levels, in the form of auditing, than their male colleagues. (Abbassi et al., 2021) conducted Research stated that the large board of directors enhances stock liquidity because board members play vital monitoring role to reduce information asymmetry and eventually helping to enhance stock liquidity.

Mbanyele (2021) argued that board networks enhances stock liquidity more *via* the information channel when economic policy uncertainty is high. Furthermore, companies with more effective boards forecast higher profits and generate more accurate projections. Consequently, lower information asymmetry should be linked to higher board efficiency. In line with the above discussion, the following hypothesis has been developed.

*H1*: The board monitoring index has a positive relationship with stock liquidity.

### Ownership concentration and stock liquidity

Ownership concentration can cause an agency problem between informed stockholders and uninformed stockholders. Informed stockholders can get the benefit of insider information. Minority stockholders can be expropriated by majority stockholders to protect their personal interest by using the corporate recourses. Agency problems are occurred due to the conflict of minority and majority stockholders which leads to agency cost, and it can influence stock market in those economies where corporate governance is really weak (La Porta et al., 2002).

Hunjra et al. (2020) Stated that ownership concentration significantly affects stock liquidity. Leaño and Pedraza (2018) documented that ownership concentration inversely significant influence stock liquidly. The empirical evidence remains far from conclusive on these theoretical claims. This problem has been examined in a variety of studies on U.S. stocks (Sarin et al., 1999; Dennis and Weston, 2002). In developing countries such as Pakistan, concentrated ownership is prevalent. For these reasons, findings based on developed countries cannot be extended to emerging markets. Hence, following the above discussion, it is hypothesized that:

*H2*: Concentrated ownership is negatively related to stock liquidity.

### Institutional ownership and stock liquidity

Generally, institutional owners are considered to be the very important player for stock liquidity. Cao and Petrasek (2014) observe substantial and positive connections between institutional investors and stock liquidity. The effect of institutional ownership on stock liquidity is different from the effect of an individual shareholder. The stock of individual investors is less liquid than that of institutional investors, as individual investors are much more driven by sentiment than institutional investors. Baker and Stein (2004) note that institutional investors are able to improve liquidity and thereby lower the risks associated with stock liquidity.

institutional investors are encouraged to get social as well as financial returns (Dyck et al., 2019). Khorana et al. (2005) stated that institutional owners are considered to be important in developed economies as it is gaining impotence now in developing economies rapidly. Dang et al. (2018) stated that there is positive significant relationship between institutional ownership and stock liquidity. Ali and Hashmi (2018) also argued that institutional investors lead stock liquidity. According to the above literature institutional investors plays an important role to enhance stock liquidity. This study predicts a significant relationship between institutional ownership and stock liquidity and it is hypothesized that:

*H3*: Institutional ownership is positively related to stock liquidity.

### Audit committee characteristics and stock liquidity

The audit committee is regarded as the most qualified body for the management of monetary information. Poor management may pose a risk to stock liquidity, affecting shareholders in the market through the influence of asymmetric information. The independence of audit committee members can play a key role to influence the efficiency of the audit committee n supervising the financial reporting procedure. To release the supervising role and defend the owner’s interest (Dwekat et al., 2020). Agency theory suggests that auditing provides a means to reduce information asymmetry, overcoming interest discrepancies between agent and principal and reducing related agency costs (Jensen and Meckling, 1976; Clinch et al., 2012).

Prior studies suggests that the committee’s efficiency is improved with a large audit committee, reduces information asymmetry and increases the quality of MRR financial reporting and, which enhances investor confidence and improve stock market liquidity (Al-Jaifi et al., 2017; Appuhami and Tashakor, 2017). Following the above literature, it is hypothesized that:

*H4*: The audit committee index has a positive relationship with stock liquidity.

### Corporate governance index and stock liquidity

Biswas (2020) claims that an increase in corporate governance quality will enhance stock liquidity. Other studies use the quality of internal corporate governance to assess stock liquidity. Coffee (1991) asserts that major investors endorse internal governance structures because these mechanisms boost stock liquidity, rendering the investors’ exit less costly. Khyareh and Amini (2021) studied the relation between governance quality, entrepreneurship and economic growth and found that there is significant impact of entrepreneurship and governance on economic growth.

Strengthened management is expected to reduce information asymmetry, which in turn improves organizational transparency (Leuz et al., 2003; Durana et al., 2021). Goh et al. (2015) indicate that the relationship between governance and liquidity is primarily influenced by managers’ effectiveness in minimizing the problems of institutions, including insider dealing and limited disclosure. However, agency issues between controlling shareholders and minority shareholders are more critical in developing economies than in developed markets (Claessens et al., 2000; Faccio and Lang, 2002; Allen, 2005).

In developing countries such as Pakistan, corporate governance tends to be weaker than in developed countries. For this reason, findings based on developed countries cannot be extended to emerging markets. Moreover, evidence from developed countries is mixed and inconclusive, with a range of distinctive features. Therefore, the following hypothesis is developed.

*H5*: Corporate governance quality is positively related to stock liquidity.

### Corporate governance, financial transparency, and stock liquidity

The relationship between stock returns and liquidity was analyzed by (Nguyen et al., 2021) the authors pointed out the significant association between expected income and liquidity of equity by using Amihud illiquidity. Liquidity is the ability to trade fast and with rates that are not substantially moving and lead to economic growth (Schwartz et al., 2020). Glosten and Milgrom (1985) find that corporate governance practices affecting liquidity are associated with a risk, Shareholder may face under conditions of asymmetrical information. In general, the literature suggests that corporate governance has a positive effect on stock liquidity, specifically that corporate governance and stock liquidity have a significant and positive relationship. These studies suggest that superior corporate governance improves stock liquidity [see, for example, (Mangena and Tauringana, 2007; Chung et al., 2010; Tang and Wang, 2011; Prasanna and Menon, 2012; Mitan et al., 2021; Arazpour and Fadaeinejad, 2014)].

Sosnowski (2022) Studied the persistence firm’s earning reports in the process of IPO, and they found that there is higher persistence in the pre-IPO earnings as compared to the year of IPO earnings. The authors studied real earning management and the use of accrual to inflate revenue and earnings. They found that discretionary accruals are used by managers of the newly listed firms to decrease cost of production, discretionary expenses and abnormal cash flow (Sosnowski, 2021). Brabenec et al. (2020) studied the difference between common and preferred stock in Europe stock market and find that current traded stock does not have characteristics which said typically, they have comparable risk as common stock do. The authors studied the nexus between corporate financial stability and.

earnings management and found that due to the threat of bankruptcy the firm manipulate their earnings to maintain competitiveness and credibility (Valaskova et al., 2021).

Utami et al. (2020) pointed out that ownership structure significantly affects stock liquidity. A perfect example is a description by Sir Adrian Cadbury of corporate governance as the structure that regulates and governs businesses. As discussed above, better corporate governance enhances stock liquidity, and financial transparency is also positively related to stock liquidity (Jain et al., 2008). As Pakistan has a relatively weak corporate governance environment, most of the firms in this country seek to enhance stock liquidity *via* financial transparency.

To the best of our knowledge, no studies have examined the moderating effect of financial transparency on the relationship between corporate governance and stock liquidity. This study is intended to fill this gap in the literature. In accordance with the substitution hypothesis of (La Porta et al., 2000), it is hypothesized that:

*H6*: Financial transparency has significantly negative effect on the relationship between corporate governance and stock liquidity in Pakistan.

## Data and research design

### Sample

The sample for this study consists of all of the non-financial listed firms on the Pakistan stock exchange during the 2009–2019 period. Financial firms are excluded due to this industry’s unique regulatory requirements and accounting procedures. The initial sample consisted of 6,930 firm-year observations. After omitting firms with missing stock prices and corporate governance data. Firms having less than 3 years data were retained in the sample. The study obtains a final sample size of 2,466 firm-year observations of 230 firms for the 2009–2019 period as reported in the Table 1. The sample above is well according to related studies, e.g., (Biswas, 2020; Xu and Liu, 2020).

**Table 1:** Sample distribution.

**S.No**	**Criteria**	**Firm-year observations**
1	Initial data	6,930
2	After excluding firms with missing stock price data	6,103
3	After excluding years not having CG data	2,728
4	After excluding firms having less than 3-Years data	2,466
5	Finally, 230 non-financial listed firms at PSX for 2009–19	2,466

### Why Pakistan?

The prime motivation of this study is to connect two vital areas of the finance literature: market microstructure and corporate finance. These two major areas have been developed separately but very rarely examined together. The Study linked these two areas by analyzing the effect of corporate governance on stock liquidity. The study also focus on the moderating role of financial transparency on the relation between corporate governance and stock liquidity, a topic not addressed in other studies. It is essential to understand how corporate strategies affect microstructure, as this can help monitors to design suitable trade regulations and help shareholders and investors to set comprehensive strategies for their stock trading (Table 2).

**Table 2:** Variable descriptions.

Variable	Abbreviation	Measurement
**Dependent variables (Stock liquidity) Price impact frequency**		
Amihud illiquidity estimate	Amihud	Daily ratio of absolute stock return to trading volume in Pakistani rupees averaged over the number of trading days in the financial year.
Liquidity Ratio	Amivest	Sum of daily trading volume over the sum of absolute stock return in a year.
Trading frequency		
Turnover-adjusted zero daily volumes	LM	Turnover-adjusted zero daily volumes
Trading cost		
Zero return measure	Zero	Proportion of zero daily returns over number of trading days in the financial year
**Independent variables**		
Corporate governance index	CG_index	Combined index of BOD, GD, AC, OC.
Board of directors	Board_index	1) Board independence.
		2) CEO duality
		3) Board size
		4) Board meeting
		5) Gender diversity
Audit committee	Audit_index	1) Audit committee size
		2) Audit committee meeting
		3) Audit committee independence
		4) Engagement of Big 4 auditors
Ownership concentration	Top_Own	1) Shares of largest shareholder divided by total number of outstanding shares
Institutional ownership	Inst_Own	1) Shares owned by institutions divided by total number of outstanding shares
**Moderator**		
Financial Transparency	FT	Earnings aggressiveness, loss avoidance and earnings smoothing.
**Control variables**		
Firm size	size	Number of shares outstanding times share price at the end of fiscal year.
Leverage	leverage	Book value of total liabilities over book value of total assets.
Firm age	Age	The year, firm registered at the PSX.
Stock price	S_price	Natural log of stock price.
Volatility	VOLATILITY	Standard deviation Daily stock return.

Source: Author’s calculations (2020)

Studies by (Chung et al., 2010; Ahmed and Ali, 2017) that directly analyze the relationship between corporate governance and stock liquidity are based solely on developed markets in the U.S. and Australia. Due to regulatory and institutional differences, it is not clear whether their results can be generalized to countries in which the market is not as developed. Emerging markets such as Pakistan’s represent a significant alternate setting to analyze this problem, for multiple reasons.

First, Pakistan has highly concentrated business ownership, with firms mostly held by families. The corporate boards of such organizations act as a rubber stamp and one family holds the bulk of the shares. Such companies are owned by individuals, the state, and international executives, and these stakeholders all actively participate in the companies’ affairs and weaken the objectivity and discretion of the board.

Second, Pakistani firms rely mostly on loans from banks as a major source of financing; thus, capital market financing plays a more passive role than in developed markets. As Pakistani firms rely much less on capital market financing, stock liquidity also plays a different role in Pakistan. Third, the public capital market is not as developed as in the U.S. and displays weak financial transparency. This causes information asymmetry and problems of adverse selection, resulting in a significantly less liquid market. More specifically, Pakistan’s financial markets are not sophisticated and have yet to gain the level of information transparency found in developed markets. Its financial analysts do not provide the same level of information to investors, rendering it difficult for investors in Pakistan to depend on information disclosed directly by firms.

An information environment depends on the quality of corporate governance (Leuz et al., 2003; Chung et al., 2010). The importance of corporate governance is made clear by the introduction of the first corporate governance code by the Security and Exchange Commission of Pakistan (SECP) in March 2002. Which was revised subsequently in 2013 & 2017. The role of corporate governance in increasing transparency and enhancing stock liquidity is even more critical in Pakistan than in developed economies. Due to these characteristics, Pakistan provides an ideal setting to analyze the effect of corporate governance on stock liquidity and the moderating role of financial transparency.

#### Variable measurement

#### Stock liquidity

##### Zero return measure

“Zero return measure” (also known as “null return estimate”) refers to the number of zero daily returns days reported in a year. Lesmond et al. (1999) demonstrate that the null return estimate is positively related to the spread measures, which is consistent with the cost effect of purchases on inventory returns. This measure is calculated as follows:


(1) $$zero_{it=}\frac{ZR_{it}}{TD_{it}}$$

where ZR_it_ is the number of zero-return day in year t for company i, and TD_it_ is the number of business days in year t for company i. A higher value indicates lower stock liquidity.

##### Amihud illiquidity estimate

The actual return on trading in Pakistani rupees (Amihud illiquidity estimate, ILLIQ) is measured as the total stock return amount accumulated in several business days during the financial year. It measures the extent to which the actual stock price fluctuates with volume of trading, as follows:


(2) $$ILLIQ_{it=}{\frac{1}{D}}_{t}\overset{D_{iy}}{\underset{d=1}{\sum }}\frac{\left|R_{itd}\right|}{VOLD_{itd}}$$

where idt represents the absolute stock return of company i on the d of year t, VOLD_idt_ is the volume of company i on the d of year t, and D_iy_ is the number of days available for company i on the d of year t. As ILLIQ rises, stock liquidity declines.

##### Liquidity ratio (AMIVEST)

The liquidity ratio (AMIVEST) is measured as the volume of trading linked to a stock price change unit. This is used in a number of studies (Amihud et al., 1997; Berkman and Eleswarapu, 1998; Datar et al., 1998; Krulicky and Horak, 2021). It is measured as follows:


(3) $$AMIVEST_{it=}\underset{t}{\sum }VOL_{it/}\underset{t}{\sum }\left|R_{it}\right|$$

where the limit is exchanged and where the average total stock returns are, respectively, for VOL_it_ and in the year t.

##### Turnover-adjusted zero daily volume

W. Liu (2006) proposes a new measure of stock liquidity, namely the sales-adjusted zero daily volume (LM). LM focuses on the trading speed; however, it does capture several liquidity dimensions. It is calculated as follows:


(4) $$LM_{it=}\left[NoZV_{it}+\frac{1/\left(turnover_{it}\right)}{Deflator}\right]\;\times \frac{252}{NoTD_{it}}$$

where NoZV_it_ is the number of zero day volumes for company *i* in year *t*; turnover (T) is the inventory of company *i* in year *t*; NoTD_t_ is the total number of days of trading in year *t*; and deflators are set at 480,000 (W. Liu, 2006). The NoTD element multiplication t standardization makes LM equal over time and thus standardizes trading days within 1 year. A greater LM value indicates lower liquidity.

#### Corporate governance

This study measures the influence of corporate governance on stock liquidity in Pakistani listed firms. Therefore, The corporate governance index is established by using PCA. To develop the corporate governance index, the study used board of directors’ characteristics, ownership concentration, institutional ownership, and audit committee characteristics (Bulathsinhalage and Pathirawasam, 2017; Biswas, 2020) the details are reported in Table 2.

#### Financial transparency

The moderator variable of the study is calculated using three proxies, i.e., earnings aggressiveness (EA), loss avoidance (LA), and earnings smoothing (ES; Zhou et al., 2018; Nair et al., 2019; Durana et al., 2021), as detailed below.

Earnings aggressiveness is measured by following (Qian et al., 2015):


(5) EA=(ΔTA−ΔCL−ΔCASH+ΔSTD−DEP+TP)/LTA

where EA is earnings aggressiveness, ΔTA is change in total assets, ΔCL is total current liability change, ΔCASH is change in cash flow from operation, ΔSTD is change in short term debt, DEP is depreciation expense, TP is taxes payable, and LTA is lag of total assets.

Loss avoidance is calculated by using a dummy variable defined as 1 if the profitability is 0–2%, and 0 otherwise (Carey and Simnett, 2006; Menon and Williams, 2004).

Earnings smoothing is measured by following the formula (Myers et al., 2007; Valaskova et al., 2021) and (Leuz et al., 2003):


(6) ES=STDNISTDOCF

STDNI is the standard deviation if net income and STDOCF is standard deviation of cashflow from operation.

## Econometric techniques

First, ordinary least squares (OLS) is used to check the association of corporate governance quality with four stock liquidity dimensions. The standard errors are classified as heteroscedastic and internal residual correlation by company (Petersen, 2009).

The study also uses the two-stage least squares (2SLS) method to address the problem of reverse causality. The study uses two instrumental variables. Following Prommin et al. (2014) and Ali (2016), the study uses the Corporate Governance Act of 2013 (CG_Act) as the first instrumental variable. It is a binary variable equal to 0 for any year before 2013 and 1 for year after 2013. Second, following (Yang and Zhao, 2014; Liu et al., 2015; Jiraporn et al., 2016) the study has used the second instrumental variable the industrial corporate governance index (Indus_CG_Index), which is the average industrial governance index, calculated as follows: (industry governance index – firm governance level index/total observation in industry – 1). The study first developed independent index for board characteristics and audit committee characteristics and used OLS and the 2SLS method for replication of the study, furthermore the study developed an independent index *via* PCA and regressed OLS method, and to address endogeneity problem alternate proxies for stock liquidity is used.

### Research model

The following baseline models are used to test whether the quality of a firm’s governance has any impact on stock liquidity.

To test H1, the study uses the following regression model:


(i) $$SL_{it=\beta_{0}+\beta_{1}BOD+CONTROLS+{Є}_{it}}$$

To test H2, the study uses the following regression model:


(ii) $$SL_{it=\beta_{0}+\beta_{1}OC_{it}+CONTROLS+{Є}_{it}}$$

To test H3, the study uses the following regression:


(iii) $$SL_{it=\beta_{0}+\beta_{1}inst\;Own_{it}+CONTROLS+{Є}_{it}}$$

To test H4, the study uses the following regression:


(iv) $$SL_{it=\beta_{0}+\beta_{1}AC_{it}+CONTROLS+{Є}_{it}}$$

To test H5, the study uses the following regression:


(v) $$SL_{it=\beta_{0}+\beta_{1}CGI_{it}+CONTROLS+{Є}_{it}}$$

To test H6, the study uses the following regression:


(vi) $$SL_{it=\beta_{0}+\beta_{1}CGI_{it}+\beta_{2}FT_{it}+\beta_{3}FT_{it}\times CGI_{it}+CONTROLS+{Є}_{it}}$$

where SL_it_ is the liquidity measure and CGI_it_ is the CGI measure for firm *i* in period *t*. BOD_it_ indicates board of directors, AC_it_ denotes audit committee, OC_it_ stands for ownership concentration, and FTi denotes financial transparency.

## Results and discussion

### Descriptive statistics

Descriptive statistics (were used to analyze the data, and Pearson’s correlation by following (Tijani et al., 2021; Valaskova et al., 2021). Table 3 shows the descriptive statistics for all of the measures of stock liquidity, i.e., Amihud illiquidity estimate (Amihud), liquidity ratio (AMIVEST), zero return measure (Zero) and turnover-adjusted zero daily volume. It also presents descriptive statistics for the independent, moderator, and control variables.

**Table 3:** Descriptive statistics.

**Variables**	**Observations**	**Mean**	**Std. Deviation**	**Min**	**Max**
Amihud	2,485	0.00153	0.00868	1.11e-09	0.189
Zero	2,485	0.100	0.133	0	0.944
Amivest	2,394	1.005e+09	9.991e+09	0	4.363e+11
LM	2,423	17.83	157.3	1.11e-07	297
B_Size	2,465	2.066	0.166	1.609	3.045
B_Indepeendce	2,465	0.175	0.188	0	1
B_Meeting	2,407	1.639	0.316	0	3.497
B_Diversity	2,465	0.0945	0.139	0	1
CEO_Duality	2,466	0.172	0.377	0	1
Board_Index	2,407	−7.75e-09	1.000	−0.674	6.540
Audit_Size	2,463	1.195	0.179	0.693	2.079
Audit_Meeting	2,428	1.421	0.124	0	2.485
Audit_Indep	2,463	0.164	0.182	0	1
Big_4	2,466	0.451	0.498	0	1
Audit_Index	2,426	5.28e-09	1.000	−0.907	1.102
Inst_Own	2,462	0.106	0.128	0	0.895
CG_Index	2,389	1.15e-08	1.000	−2.747	5.886
Loss_Avoid	2,525	0.139	0.346	0	1
E_Aggres	2,177	0.0526	0.318	−0.858	8.408

Source: Author’s Calculation (2020)

Amihud is calculated as the ratio of daily absolute stock return to volume, in Pakistani rupees, averaged over the number of trading days in the financial year. The mean value for Amihud is 0.00153, with a standard deviation value of 0.00868. The minimum Amihud value is 1.11e-09, and the maximum value is 0.18. Zero is calculated as the number of zero-return days divided by trading days. The mean value of Zero is 0.100, ranging from a minimum value of 0 to a maximum value of 0.944, with a standard deviation of 0.133.

### Board characteristics and stock liquidity

The study uses PCA to develop a board index. The main objective of PCA is to decrease the number of variables in uncorrelated mechanisms. Incorporation of the largest variance of data is the first step of a PCA. The largest variance factors for the representation of board independence, board size, board meetings, board diversity, and CEO duality were selected as suggested by Tarchouna et al. (2017), as shown in Appendix I.

Table 4 shows a negative relationship between the board monitoring and Amihud. This indicates that an increase in the board index causes a decrease in Amihud, which enhances stock liquidity as suggested by Amihud and Mendelson (1986). The results affirm hypothesis 1 of the study that, due to board monitoring information asymmetry and agency problems are reduced which causes increase in stock liquidity. These results are in line with Ali et al. (2017).

**Table 4:** Board index and stock liquidity (OLS Regression).

**Variables**	**Amihud**	**Amihud**	**LM**	**LM**
Board_Index	−0.000323**	−0.000337**	10.79***	11.27***
	(−2.076)	(−2.111)	(3.195)	(3.240)
Leverage	−0.00119**	−0.00129**	14.83	19.00
	(−2.288)	(−2.340)	(1.311)	(1.579)
Size	−0.000164*	−0.000200	−8.559***	−6.408**
	(−1.727)	(−1.546)	(−4.145)	(−2.282)
Age	0.000370	0.000444	1.821	6.062
	(0.937)	(1.012)	(0.212)	(0.635)
S_Price	−8.07e-05	−0.000132	7.720***	8.814***
	(−0.631)	(−0.860)	(2.775)	(2.649)
Volatility	0.0795***	0.0788***	−26.09	−55.21
	(29.05)	(27.99)	(−0.439)	(−0.902)
Constant	0.000581	0.000995	157.4***	73.96
	(0.230)	(0.307)	(2.863)	(1.048)
Observations	2,348	2,348	2,348	2,348
R-squared	0.318	0.325	0.015	0.025
Industry FE	No	Yes	No	Yes

*t*-Statistics in parentheses.

*** *p* < 0.01;

** *p* < 0.05;

* *p* < 0.1.

The relationship between the board index and Amihud remains the same after controlling for industry fixed effects. As seen in Table 4, there is a positive relation between the board monitoring and LM. This is inconsistent with the literature; however, this may be due to an endogeneity problem.

Table 5 shows the 2SLS results. In the first stage, the board index is regressed, which is a self-developed index, *via* PCA, including six board measures. The instrumental variables, Indus_CG_Index and CG_Act, are defined above.

**Table 5:** Board index and stock liquidity (2SLS) Estimation.

**Variables**	**1**_**st**_**stage Board_index**	**2** ^**nd**^ **stage Amihud**	**2** ^**nd**^ **stage LM**
Board_index		−0.0224***	−36.69**
		(−3.079)	(−2.379)
Indus_B_index	−0.0857		
	(−1.364)		
CG_Act	0.133***		
	(3.019)		
Leverage	−0.189***	−0.00500**	−1.642
	(−2.710)	(−2.426)	(−0.374)
size	−0.100***	−0.00224***	−8.778***
	(−7.871)	(−3.014)	(−5.631)
age	−0.339***	−0.00736***	−11.38*
	(−6.477)	(−2.597)	(−1.897)
S_price	0.0127	0.000340	5.545***
	(0.732)	(0.800)	(6.117)
Volatility	0.309	0.0872***	77.53***
	(0.846)	(9.791)	(4.059)
Constant	3.348***	0.0735***	217.4***
	(10.10)	(2.911)	(4.098)
Observations	2,318	2,318	2,307
R-squared		0.0541	0.197
Industry FE	No	No	No

*t*-Statistics in parentheses.

*** *p* < 0.01;

** *p* < 0.05;

* *p* < 0.1.

The table shows a significant, negative association between the board index and Stock liquidity (Amihud) at the 1% level, which means that an increase in the board index results in an increase in stock liquidity. This supports H1, which predicts that board characteristics are positively related to stock liquidity. Tables 4 and 5 show a significant negative relationship between the board index and LM, which indicates that a decrease in LM leads to an increase in stock liquidity. The findings of the study affirm the hypothesis which states that strong board monitoring will decrease agency problems which results in high stock liquidity.These results are in line with (Ali et al., 2017).

### Audit committee characteristics and stock liquidity

The study used PCA to develop an audit quality index. The largest variance for the representation of audit independence, audit size, audit committee meetings and Big 4 auditor engagement is selected, as suggested by (Tarchouna et al., 2017; See Appendix II).

Table 6 presents the results of the OLS for the audit index and stock liquidity proxies (Amihud and LM) with the audit committee characteristics index, established *via* PCA, including audit committee independence, audit committee size, audit committee meeting and a dummy variable to indicate whether the firm was audited by a Big 4 audit firm.

**Table 6:** Audit committee index and stock liquidity (OLS Regression).

**Variables**	**Amihud**	**Amihud**	**LM**	**LM**
Audit_Index	0.000239**	0.000246**	−3.818***	−1.864**
	(2.384)	(2.104)	(−5.581)	(−2.232)
Leverage	−0.00113	−0.00120	4.788**	6.631***
	(−1.531)	(−1.497)	(2.314)	(3.001)
size	−0.000194*	−0.000202	−4.463***	−5.013***
	(−1.836)	(−1.535)	(−9.804)	(−8.931)
age	0.000452	0.000557	0.857	2.384
	(1.450)	(1.561)	(0.742)	(1.602)
S_Price	−7.80e-05	−0.000128	4.979***	6.267***
	(−0.747)	(−0.901)	(9.072)	(8.836)
Volatility	0.0797***	0.0789***	67.70***	60.47***
	(4.749)	(4.637)	(4.413)	(3.993)
Constant	0.000885	0.000547	79.21***	68.92***
	(0.360)	(0.181)	(7.867)	(5.873)
Observations	2,366	2,366	2,355	2,355
R-squared	0.318	0.324	0.121	0.160
Industry FE	No	Yes	No	Yes

Robust *t*-statistics in parentheses.

*** *p* < 0.01;

** *p* < 0.05;

* *p* < 0.1.

A high score on the audit index indicates high corporate governance quality. The study also control for firm size, firm age, volatility, and stock price. After controlling for industry fixed effects, the results were same. The Table 6 shows a positive association between the audit index and Amihud, which is inconsistent with the literature. This may be due to endogeneity issues.

The study has used control variables, i.e., firm size, firm age, leverage, volatility, and stock price. The first instrumental variable is industrial audit index average (Indus_A_index) and the second instrumental variable is CG_Act. As shown in the Table 7, both of the instrumental variables are significant at the 1% level, which indicates that the instrumental variables are strong.

**Table 7:** Audit committee index and stock liquidity (2SLS Estimation).

	**First**	**2nd_stage**	**2nd_stage**
**Variables**	**Auidt_index**	**Amihud**	**LM**
Audit_Index		−0.00265***	−9.243***
		(−4.565)	(−4.341)
Indus_A_Index	0.444***		
	(13.61)		
CG_Act	−0.241***		
	(−6.548)		
Leverage	−0.220***	−0.000752	3.961*
	(−3.708)	(−1.342)	(1.837)
size	0.155***	−0.000721***	−3.372***
	(13.28)	(−4.515)	(−6.003)
age	0.0319	0.000323	1.791
	(0.733)	(0.787)	(1.509)
S_Price	0.0470***	−0.000125	5.070***
	(3.260)	(−0.929)	(9.045)
Volatility	0.454	0.0795***	67.76***
	(1.486)	(27.69)	(4.409)
Constant	−3.340***	0.0126***	52.89***
	(−11.31)	(3.284)	(4.222)
Observations	2,335	2,335	2,324
R-squared		0.262	0.104
Industry FE	No	No	No

Robust *t*-statistics in parentheses

*** *p* < 0.01;

** *p* < 0.05;

* *p* < 0.1.

The results show a significant, negative relation of Audit committee index with LM, which indicates that a decrease in LM increases stock liquidity, as suggested by (W. Liu, 2006). The findings of the study affirms hypothesis 2, and states that strong audit committee and audit by big four firms reduces information asymmetry and decreases frauds which leads to higher stock liquidity (Searat Ali et al., 2017; Biswas, 2020).

### Ownership concentration and stock liquidity

Table 8 confirms a positive relationship between ownership concentration and stock liquidity (Amihud), which is in line with (Searat Ali et al., 2017). These results remain consistent after controlling for industry fixed effects. The table also shows the results of the regression of ownership concentration and stock liquidity, and control for leverage, firm size, firm age, stock price, volatility, and industry fixed effect.

**Table 8:** Ownership concentration and Stock liquidity (OLS Regression).

**Variables**	**Amihud**	**Amihud**	**LM**	**LM**	
Top5_Own	0.228**	0.327***	1.787	2.217
	(2.494)	(3.333)	(0.536)	(0.641)
Leverage	−0.00114	−0.00122	4.646**	6.468***
	(−1.633)	(−1.622)	(2.267)	(3.008)
Size	−0.000139	−0.000165	−5.332***	−5.253***
	(−1.322)	(−1.307)	(−11.40)	(−9.309)
Age	0.000440	0.000575*	0.572	2.102
	(1.461)	(1.680)	(0.478)	(1.388)
S_Price	−7.63e-05	−0.000107	4.925***	6.165***
	(−0.733)	(−0.762)	(9.058)	(8.903)
Volatility	0.0786***	0.0781***	65.44***	55.91***
	(4.728)	(4.635)	(4.250)	(3.716)
Constant	−0.000352	−6.39e-05	100.5***	74.01***
	(−0.147)	(−0.0223)	(9.926)	(6.380)
Observations	2,404	2,404	2,392	2,392
R-squared	0.312	0.319	0.109	0.158
Industry FE	No	Yes	No

Robust *t*-statistics in parentheses.

*** *p* < 0.01;

** *p* < 0.05;

* *p* < 0.1.

then estimated the model after controlling for endogeneity using 2SLS. This study uses an instrumental variable defined as the median of the top five ownership concentrations in the industry (median_T5), as industry-level ownership is expected to affect firm-level ownership but not be related to the influence of industry ownership on stock liquidity.

The second instrumental variable is CG_Act. Table 9 shows the positive relation between the audit index and Amihud. It suggests that firms with concentrated ownership are higher alignment and have lower level of agency conflict. Therefore, they are concerned with high stock liquidity. The results are parallel to Ali et al. (2021).

**Table 9:** Ownership concentration and stock liquidity (2SLS Estimation).

	**First**	**2** ^**nd**^ **_Stage**	**2** ^**nd**^ **_Stage**
**Variables**	**Top5_Own**	**Amihud**	**LM**
Top5_Own		0.532*	55.17***
		(1.953)	(6.382)
median_T5	0.678***		
	(16.55)		
CG_Act	−0.0122		
	(−1.432)		
Leverage	0.0648***	−0.00113**	9.175***
	(4.747)	(−2.053)	(3.883)
size	0.00682***	−0.000137	−4.711***
	(2.769)	(−1.432)	(−9.804)
age	0.0224**	0.000444	2.233*
	(2.205)	(1.137)	(1.691)
S_Price	0.00856**	−7.43e-05	5.698***
	(2.535)	(−0.573)	(9.332)
Volatility	0.245***	0.0786***	79.24***
	(3.439)	(28.47)	(4.866)
Constant	−0.108	−0.000327	109.8***
	(−1.628)	(−0.133)	(9.680)
Observations	2,404	2,404	2,392
R-squared		0.312	0.002
Industry FE	No	No	No

Robust *t*-statistics in parentheses.

*** *p* < 0.01.

** *p* < 0.05.

* *p* < 0.1.

### Institutional ownership and stock liquidity

Table 10 shows the positive association between institutional ownership and both measures of stock liquidity. After controlling for industry fixed effects, the same significant results were found. and support the hypothesis of the study that institutional ownership is positively related to stock liquidity. The results affirm the hypothesis and states that high institutional ownership decreases information asymmetry and agency problems and which enhances stock liquidity in Pakistani listed firms. The results are parallel to (Searat Ali et al., 2017). The small R-squared in the relevant field of accounting is consistent with prior studies sech as (Searat Ali et al., 2017; Biswas, 2020).

**Table 10:** Institutional ownership and stock liquidity (OLS Regression).

**Variables**	**Amihud**	**Amihud**	**LM**	**LM**
Inst_Own	−0.00174*	−0.00194*	−13.35***	−12.53***
	(−1.739)	(−1.736)	(−3.074)	(−2.882)
Leverage	−0.00112	−0.00122	4.462**	6.511***
	(−1.569)	(−1.589)	(2.163)	(3.012)
Size	−0.000149	−0.000161	−5.260***	−5.274***
	(−1.466)	(−1.277)	(−11.49)	(−9.364)
Age	0.000418	0.000537	0.732	2.337
	(1.374)	(1.556)	(0.619)	(1.561)
S_Price	−6.45e-05	−0.000118	4.837***	6.254***
	(−0.629)	(−0.849)	(9.007)	(9.001)
Volatility	0.0788***	0.0781***	64.17***	56.11***
	(4.743)	(4.646)	(4.177)	(3.721)
Constant	−0.000162	−0.000340	99.11***	75.78***
	(−0.0680)	(−0.118)	(9.853)	(6.538)
Observations	2,403	2,403	2,391	2,391
R-squared	0.313	0.320	0.112	0.160
Industry FE	No	Yes	No	Yes

Robust *t*-statistics in parentheses.

*** *p* < 0.01;

** *p* < 0.05;

* *p* < 0.1.

### Corporate governance and stock liquidity

The study regress the stock liquidity proxies, Amihud and LM, with Corporate Governance index. The results shown in Table 11 reveal that corporate governance is related to stock liquidity. Specifically, firms with strong corporate governance have higher stock liquidity. The results support H5 and are consistent with Ali et al. (2017).

**Table 11:** Corporate governance index and stock liquidity (OLS Regression).

**Variables**	**Amihud**	**Amihud**	**LM**	**LM**
CG_Index	−0.000263**	−0.000252**	−0.849*	- 4.4633**
	(−2.362)	(−2.007)	(−1.650)	(−2.031)
Leverage	−0.00118	−0.00129	5.623***	7.394***
	(−1.579)	(−1.591)	(2.660)	(3.332)
size	−0.000195*	−0.000203	−5.094***	−5.150***
	(−1.870)	(−1.581)	(−10.58)	(−9.039)
age	0.000387	0.000500	1.161	2.527*
	(1.175)	(1.313)	(0.986)	(1.677)
S_Price	−6.70e-05	−0.000130	4.788***	6.091***
	(−0.633)	(−0.904)	(8.766)	(8.801)
Volatility	0.0796***	0.0788***	64.32***	56.60***
	(4.721)	(4.620)	(4.179)	(3.753)
Constant	0.00112	0.000807	91.83***	71.65***
	(0.449)	(0.268)	(8.617)	(5.982)
Observations	2,332	2,332	2,321	2,321
R-squared	0.318	0.325	0.113	0.159
Industry FE	No	Yes	No	Yes

Robust *t*-statistics in parentheses.

*** *p* < 0.01;

** *p* < 0.05;

* *p* < 0.1.

Table 12 shows the 2SLS results. In the first stage CG_index is regressed. The instrumental variable is CG_Act. While Amihud and LM are used as liquidity measures. The table shows a significant, negative relation between CG_index and Amihud at the 1% level. This confirms that good governance increases stock liquidity. The table depicts a negative relation between CG_index and LM. the findings states that strong corporate governance quality will decrease information asymmetry and agency problems and save monitory shareholders from expropriation which leads to high stock liquidity in Pakistani listed firms. The results are consistent with (Ali et al., 2017; Biswas, 2020) and are not subject to endogeneity. The results support H5: good corporate governance enhances stock liquidity.

**Table 12:** Corporate governance index and stock liquidity (2SLS Estimation).

	**First**	**2** ^**nd**^ **stage**	**2** ^**nd**^ **stage**
**Variables**	**CG_Index**	**Amihud**	**LM**
CG_Index		−0.00500***	−9.243***
		(−3.705)	(−4.341)
Indus_CG_Index	0.237***		
	(6.031)		
CG_Act	−0.121***		
	(−2.924)		
Leverage	0.205***	−0.00221***	6.590***
	(3.070)	(−3.190)	(2.922)
size	0.178***	−0.00116***	−4.298***
	(13.89)	(−3.930)	(−5.802)
age	0.429***	−0.00160**	3.505*
	(8.693)	(−2.163)	(1.914)
S_Price	−0.0636***	0.000279	4.472***
	(−3.897)	(1.529)	(7.864)
Volatility	−0.426	0.0821***	61.77***
	(−1.241)	(24.90)	(3.912)
Constant	−5.170***	0.0284***	67.09***
	(−15.96)	(3.419)	(3.409)
Observations	2,302	2,302	2,291
R-squared		0.077	0.100
Industry FE	No	No	No

Robust *t*-statistics in parentheses.

*** *p* < 0.01;

** *p* < 0.05;

* *p* < 0.1.

### Corporate governance, financial transparency, and stock liquidity (OLS regression)

There is a negative relation between the interaction term of CG_index and financial transparency with stock liquidity, significant at the 1% level (see Table 13). The results also show a negative relationship between the interaction term of CG_index and financial transparency and stock liquidity. These results hold after controlling for industry fixed effects, as shown in Table 13.

**Table 13:** CG_index, financial transparency and stock liquidity (OLS Estimation).

**Variables**	**Amihud**	**Amihud**	**LM**	**LM**	**Amihud**	**Amihud**	**LM**	**LM**	**Amihud**	**Amihud**	**LM**	**LM**
CG_Index X. E_Aggres	0.00332**	0.00342**	2.022**	0.0443**								
	(2.017)	(2.049)	(2.185)	(2.137)								
E_Aggres	0.00454*	0.00454*	3.984	4.452								
	(1.920)	(1.926)	(1.188)	(1.383)								
Loss_Avoid X CG_Index					0.0543***	0.0508**	3.146**	2.548*				
					(3.161)	(2.358)	(2.180)	(1.764)				
Loss_Avoid					6.58e-05	0.000114	3.373*	2.421				
					(0.134)	(0.231)	(1.658)	(1.194)				
CG_Index X E_Sm3									0.0011***	0.00116***	2.903*	7.677**
									(2.609)	(2.663)	(1.952)	(2.480)
E_Sm3									0.00157**	0.00155**	0.849	−0.0732
									(2.052)	(1.976)	(0.200)	(−0.0174)
CG_Index	0.000355**	0.000355**	−0.964*	−0.278	0.000267**	0.000255*	−0.440	0.236	−0.000824*	−0.000885*	−0.165	0.751
	(2.372)	(2.243)	(−1.796)	(−0.472)	(2.242)	(1.936)	(−0.789)	(0.393)	(−1.770)	(−1.848)	(−0.0519)	(0.241)
												
Leverage	−0.000605	−0.000699	4.235**	6.025***	−0.00118	−0.00128	5.986***	7.644***	−0.00121	−0.00133	5.598***	7.393***
	(−0.693)	(−0.741)	(2.125)	(2.887)	(−1.588)	(−1.595)	(2.821)	(3.447)	(−1.602)	(−1.618)	(2.664)	(3.341)
Size	−0.000117	−0.000124	−4.455***	−4.516***	−0.000194*	−0.000201	−5.046***	−5.109***	−0.000193*	−0.000206	−5.095***	−5.150***
	(−1.010)	(−0.914)	(−8.631)	(−7.477)	(−1.813)	(−1.533)	(−10.56)	(−9.049)	(−1.851)	(−1.615)	(−10.60)	(−9.036)
Age	0.000236	0.000246	0.549	1.917	0.000383	0.000493	0.932	2.391	0.000388	0.000501	1.159	2.526*
	(0.730)	(0.667)	(0.518)	(1.379)	(1.195)	(1.331)	(0.785)	(1.569)	(1.177)	(1.316)	(0.984)	(1.676)
S_Price	−0.000170*	−0.000220	4.487***	5.830***	−6.54e-05	−0.000127	4.867***	6.134***	−6.99e-05	−0.000130	4.790***	6.092***
	(−1.770)	(−1.637)	(7.850)	(8.018)	(−0.625)	(−0.893)	(8.807)	(8.825)	(−0.661)	(−0.908)	(8.777)	(8.796)
Volatility	0.0680***	0.0674***	32.85**	23.92	0.0796***	0.0789***	64.96***	57.21***	0.0802***	0.0794***	64.62***	56.56***
	(2.997)	(2.936)	(1.968)	(1.464)	(4.705)	(4.608)	(4.200)	(3.775)	(4.700)	(4.598)	(4.152)	(3.698)
Constant	0.000211	0.000223	81.92***	61.84***	0.00110	0.000756	90.63***	70.44***	−0.000465	−0.000681	91.01***	71.75***
	(0.0732)	(0.0690)	(7.346)	(4.949)	(0.422)	(0.243)	(8.544)	(5.942)	(−0.159)	(−0.201)	(8.045)	(5.719)
Observations	2,080	2,080	2,070	2,070	2,332	2,332	2,321	2,321	2,332	2,332	2,321	2,321
R-squared	0.267	0.273	0.087	0.139	0.318	0.325	0.115	0.160	0.319	0.326	0.113	0.159
Industry FE	No	Yes	No	Yes	No	Yes	No	Yes	No	Yes	No	Yes

Robust *t*-statistics in parentheses.

*** *p* < 0.01;

** *p* < 0.05;

* *p* <0.1.

The results show that in firms with high financial transparency there is a weak association between corporate governance and stock liquidity. This relationship remains same after controlling for industry fixed effects. This means that financial transparency negatively moderates the relationship between corporate governance and stock liquidity. That is, financial transparency acts as a substitute for corporate governance in firms with weak corporate governance in Pakistan to enhance stock liquidity. These results are interpreted according to the substitution concept established by La Porta (1997).

### Corporate governance, financial transparency, and stock liquidity (2SLS regression)

Table 14 shows the 2SLS results. The instrumental variables are the industrial governance index and the interaction term of CG_index and earning aggressiveness. The table shows a significant, negative association between the interaction term of CG_index and financial transparency and all three proxies of stock liquidity. This means that financial transparency negatively moderates the relationship between corporate governance and stock liquidity. That is, financial transparency acts as a substitute for corporate governance in firms with weak corporate governance in Pakistan to enhance stock liquidity. These results are interpreted according to the substitution concept established by La Porta (1997).

**Table 14:** CG_index, financial transparency and stock liquidity (2SLS Estimation).

	**First**	**2nd Stage**	**2nd Stage**	**First**	**2nd Stage**	**2nd Stage**	**First**	**2nd Stage**	**2nd Stage**
**Variables**	**CG_Index**	**Amihud**	**LM**	**CG_Index**	**Amihud**	**LM**	**CG_Index**	**Amihud**	**LM**
CG_Index X E_Aggres		0.00584***	6.855**						
		(−6.776)	(−2.341)						
CG_Index X E_Aggres	0.0688								
	(0.802)								
E_Aggres	−0.0391	0.00490***	4.411*						
	(−0.463)	(8.411)	(1.672)						
Loss_Avoid X CG_Index					0.000938*	4.432**			
					(1.764)	(2.270)			
Loss_Avoid#c.Indus_CG_Index				0.332***					
				(3.114)					
Loss_Avoid				0.122**	5.82e-05	4.503**			
				(2.222)	(0.124)	(2.153)			
CG_Index X E_Sm3								0.00107*	5.47***
								(1.768)	(7.668)
Indus_CG_Index X E_Sm3							−0.0114		
							(−0.0502)		
E_Sm3							−0.122	0.00161	−0.970
							(−0.959)	(1.542)	(−0.209)
CG_Index		0.00104	−11.58*		0.000362	−13.84**		−0.00448	−28.74*
		(0.727)	(−1.798)		(0.243)	(−2.096)		(−1.198)	(−1.733)
Indus_CG_Index	0.215***			0.204***			0.257		
	(4.997)			(4.910)			(1.133)		
Leverage	0.168**	−0.000700	6.341**	0.195***	−0.00112*	8.671***	0.197***	−0.00117*	8.354***
	(2.363)	(−1.199)	(2.390)	(2.921)	(−1.812)	(3.153)	(2.951)	(−1.912)	(3.068)
Size	0.172***	−0.000227	−2.396*	0.176***	−0.000192	−2.565*	0.173***	−0.000185	−2.763**
	(12.69)	(−0.768)	(−1.789)	(13.80)	(−0.646)	(−1.936)	(13.61)	(−0.663)	(−2.232)
Age	0.424***	7.32e-07	5.420*	0.429***	0.000436	6.706**	0.440***	0.000458	6.714**
	(8.079)	(0.00105)	(1.724)	(8.704)	(0.618)	(2.148)	(8.939)	(0.672)	(2.237)
S_Price	−0.0705***	−0.000146	3.686***	−0.0672***	−8.51e-05	3.995***	−0.0707***	−9.38e-05	3.866***
	(−4.097)	(−0.895)	(4.970)	(−4.150)	(−0.525)	(5.507)	(−4.376)	(−0.576)	(5.337)
Volatility	−0.695	0.0683***	24.45*	−0.396	0.0795***	60.23***	−0.505	0.0802***	57.46***
	(−1.642)	(21.13)	(1.673)	(−1.150)	(28.03)	(4.774)	(−1.457)	(27.68)	(4.484)
Constant	−5.063***	0.00339	22.06	−5.197***	0.000914	18.15	−5.035***	−0.000852	24.81
	(−14.66)	(0.410)	(0.590)	(−16.02)	(0.109)	(0.488)	(−14.49)	(−0.110)	(0.727)
									
Observations	2,053	2,053	2,043	2,302	2,302	2,291	2,302	2,302	2,291
R-squared		0.252	−0.024		0.315	−0.012		0.314	−0.001
Industry FE	No	No	No	No	No	No	No	No	No

*t*-statistics in parentheses.*** *p*<0.01, ** *p*<0.05, * *p*<0.1.

### Robustness checks

This study has used Amihud and LM as measures of stock liquidity in the previous sections. As a robustness check, two alternative measures of stock liquidity: AMIVEST and Zero is used. The results remain consistent with the primary results.

The coefficients suggest that the results on the effect of board characteristics on stock liquidity do not change significantly. Thus, the results can explain the relationship between the board index and stock liquidity. The results are shown in Table IV in the Appendix.

Table V in the appendix shows the results of the robustness test for audit committee index and stock liquidity. With the 2SLS method, the results are robust for both alternative liquidity proxies, AMIVEST and Zero.

Table VI in the Appendix shows the results of the 2SLS test of the robustness of ownership concentration and stock liquidity. The results are in line with the main results.

The robustness checks on the impact of institutional ownership on stock liquidity shows that the results can explain the association of institutional ownership and stock liquidity. See Table VII in the Appendix.

The coefficients reported in Table XII in the Appendix show that the results of the analysis of corporate governance did not change under alternative proxies of stock liquidity.

The robustness test using alternative measures of stock liquidity also show that the results explaining the negative moderating role of financial transparency on the relation of corporate governance and stock liquidity are robust. These results are shown in Tables IX, X and XI in the Appendix.

## Conclusion

This study empirically analyzes the effect of corporate governance on stock liquidity and the moderating effect of financial transparency for non-financial firms listed on the Pakistan stock exchange. The study uses a sample of 230 non-financial firms listed on the Pakistan stock exchange during the 2009–2019 period to examine whether corporate governance practices affect stock liquidity in Pakistan. The study provides analytical evidence of stock liquidity in the context of agency theory and information asymmetry theory.

To the best of our knowledge, this is first study in the finance field to investigate the moderating effect of financial transparency on the relation between corporate governance and stock liquidity and the first study to analyze the relationship between corporate governance and stock liquidity in Pakistan. This is also the first study to establish new indexes for corporate governance using PCA.

The findings of the study suggest that ownership concentration has a negative effect on stock liquidity. They further suggest that institutional ownership has a positive and significant effect on stock liquidity. There is a positive and significant effect of corporate governance on enhancing stock liquidity; these results are consistent with information asymmetry theory and agency theory. The results are robust to alternative proxies of stock liquidity. The results are in line with the literature on corporate governance and stock liquidity. The findings of the study suggest that corporate governance practices are of importance to strategic choice construction and in refining financial market liquidity, as strong corporate governance enhances stock liquidity.

The study also explores the possible moderating effect of financial transparency on the relationship between corporate governance and stock liquidity in Pakistani firms. It finds a negative moderating effect of financial transparency on the relationship between corporate governance and stock liquidity. This effect remains consistent for three proxies of liquidity. These findings suggest that financial transparency may substitute for corporate governance in increasing stock liquidity, which is consistent with the substitution concept established by (La Porta et al., 2000). In Pakistan, the corporate governance environment is weak; thus, a firm’s corporate governance uses high financial transparency to improve stock liquidity.

### Limitations and future research directions

The long study period will allow the study to account for major events such as financial crises and the introduction of the nation’s first corporate governance code. The study analyzes data from only one developing country. However, taking legal and cultural differences into account, the findings can be applied to other developing economies. This study can also be performed on an international sample using panel data from multiple countries to examine the effect of individual corporate governance channels on stock liquidity. Future research could analyze the effect of disclosure quality and shareholder protection on stock liquidity. The study is conducted in developing economy Pakistan but it could of more interest if the in future the study replicate in develop economy like America & United Kingdom where corporate governance is strong, shareholders are more protected and having high financial transparency.

The findings support the suggestion that managers, corporations, and investors should be more rigorous in supervising corporate governance structures, with the goal of drafting trade laws and developing corporate environments and noise trading techniques. In addition, the study highlights the vital role audit committee independence in market liquidity. It is crucial to assess the value of this variable by specifically identifying the independent non-executive board directors in the Corporate Governance Code and that regulators pay particular attention to this information.

## Data availability statement

The raw data supporting the conclusions of this article will be made available by the authors, without undue reservation.

## Author contributions

All authors listed have made a substantial, direct, and intellectual contribution to the work and approved it for publication.

## Funding

We acknowledge the support of Research on the Long-Term Mechanism and Anti-Relative Poverty based on Property Rights protection, supported by the National Social Science Foundation of China (Grant No. 21FGLB087).

## Conflict of interest

The authors declare that the research was conducted in the absence of any commercial or financial relationships that could be construed as a potential conflict of interest.

## Publisher’s note

All claims expressed in this article are solely those of the authors and do not necessarily represent those of their affiliated organizations, or those of the publisher, the editors and the reviewers. Any product that may be evaluated in this article, or claim that may be made by its manufacturer, is not guaranteed or endorsed by the publisher.
